# Genomic and clinical characterization of a familial GIST kindred intolerant to imatinib

**DOI:** 10.1038/s41525-024-00405-z

**Published:** 2024-03-27

**Authors:** K. M. Ingley, M. Zatzman, A. M. Fontebasso, W. Lo, V. Subasri, A. Goldenberg, Y. Li, S. Davidson, N. Kanwar, L. Waldman, L. Brunga, Y. Babichev, E. G. Demicco, A. Gupta, M. Szybowska, S. Thipphavong, D. Malkin, A. Villani, A. Shlien, R. A. Gladdy, R. H. Kim

**Affiliations:** 1 Division of Medical Oncology and Hematology, Princess Margaret Cancer Centre, Toronto, Canada; 2 Department of Pediatrics, Division of Haematology/ Oncology, The Hospital for Sick Children, Toronto, ON Canada; 3 Department of Laboratory Medicine and Pathobiology, University of Toronto, Toronto, Canada; 4 Genetics and Genome Biology Program, The Hospital for Sick Children, Toronto, ON Canada; 5 Division of General Surgery, Sinai Health System, Department of Surgery, University of Toronto, Toronto, ON Canada; 6 Department of Surgical Oncology, Princess Margaret Cancer Centre, Toronto, ON Canada; 7 Department of Medical Biophysics, University of Toronto, Toronto, ON Canada; 8 Department of Computer Science, University of Toronto, Toronto, ON Canada; 9 Department of Pediatric and Laboratory Medicine, The Hospital for Sick Children, Toronto, ON Canada; 10 Department of Genetic Counselling, The Hospital for Sick Children, Toronto, ON Canada; 11 Department of Pathology and Laboratory Medicine, Sinai Health System, University of Toronto, Toronto, ON Canada; 12 Division of Medical Oncology and Hematology, Princess Margaret Cancer Centre, Fred A. Litwin Centre in Genetic Medicine, University Health Network, Toronto, ON Canada; 13 Department of Radiology, Sinai Health System, University of Toronto, Toronto, ON Canada; 14 Department of Medicine, University of Toronto, Toronto, ON Canada; 15 Present Address: Department of Oncology, Royal Children’s Hospital, Melbourne and Victorian Adolescent & Young Adult Cancer Service, Peter MacCallum Cancer Centre, Melbourne, Australia

**Keywords:** Cancer genomics, Molecular medicine

## Abstract

Familial gastrointestinal stromal tumors (GIST) are rare. We present a kindred with multiple family members affected with multifocal GIST who underwent whole genome sequencing of the germline and tumor. Affected individuals with GIST harbored a germline variant found within exon 13 of the *KIT* gene (c.1965T>G; p.Asn655Lys, p.N655K) and a variant in the *MSR1* gene (c.877 C > T; p.Arg293*, pR293X). Multifocal GISTs in the proband and her mother were treated with preoperative imatinib, which resulted in severe intolerance. The clinical features of multifocal GIST, cutaneous mastocytosis, allergies, and gut motility disorders seen in the affected individuals may represent manifestations of the multifunctional roles of *KIT* in interstitial cells of Cajal or mast cells and/or may be suggestive of additional molecular pathways which can contribute to tumorigenesis.

## Introduction

Gastrointestinal stromal tumors (GISTs) are the most common primary mesenchymal tumors of the gastrointestinal tract. Most GISTs are sporadic, but hereditary predispositions to GIST exist due to germline variants in *KIT*, *PDGFRA*, *NF1*, and *SDHx*^1–4^. In *KIT*-related familial forms of GIST, *KIT* variants frequently occur in exon 11, encoding the juxtamembrane (JM) domain^5^. Disruption of this JM domain impairs auto-inhibition and leads to uncontrolled activation of the cKIT protein^6^. The majority of these variants are substitutions, followed by deletions and insertions^3^. Germline single nucleotide variants involving the tyrosine kinase (TK) domain of the *KIT* gene, including exons 9, 13, and 17, are less frequent^7^.

The *KIT* gene is important for the development and regulation of interstitial cells of Cajal (ICC), mast cells, and melanocytes^3,8^. As a result, dysfunction of the cKIT protein has pleiotropic effects. Other variants in this gene have been documented in association with isolated mastocytosis and piebaldism (OMIM# 172800), suggesting that *KIT* variants inducing either GIST or mastocytosis may activate different downstream signaling pathways resulting in dysregulation of mast cells and/or ICC^8,9^. In addition to a predisposition for developing GISTs, individuals harboring *KIT* variants can also variably express other manifestations of disease, including changes in skin pigmentation, urticarial pigmentosa, and gut motility disturbances^4^. There can be significant variability in phenotype between affected individuals within and between families, suggestive of variable penetrance or the potential for other important contributors to these phenotypes.

The tyrosine kinase inhibitor (TKI) imatinib is first line therapy for GIST in the metastatic setting and is effective for most *KIT*-mutant GIST. The sensitivity of GIST to imatinib varies with the type of activating exon variant and its direct effect on the structure of several domains within cKIT that lead to aberrant receptor activation and altered downstream signaling^3,10^. *KIT* variants within exons 8 and 9 encode for the extracellular ligand-binding domain and stimulate stem cell factor (SCF) binding-induced activation. Variants in exon 11 encode the JM regulatory domain and allow the kinase activation loop to switch to activation, while variants within exon 13, encoding the TK1 domain, directly alter contact with the JM domain and the ATP binding pocket within *KIT*^3,10^.

We report on three generations of a family who underwent panel-based and whole genome sequencing of the tumor and germline and were found to have a gain-of-function variant in exon 13 of the *KIT* gene, c.1965T>G; p.Asn655Lys (N655K). Herein, we describe the clinical phenotype of two affected family members with GISTs and severe intolerance to imatinib in association with the p.Asn655Lys germline variant. Given the rarity of familial GIST, understanding the biological behavior of GIST kindreds and their clinical response to treatment can help us predict and tailor management.

## Results

### Clinical and pathologic description of the familial kindred

**Fig. 1:**
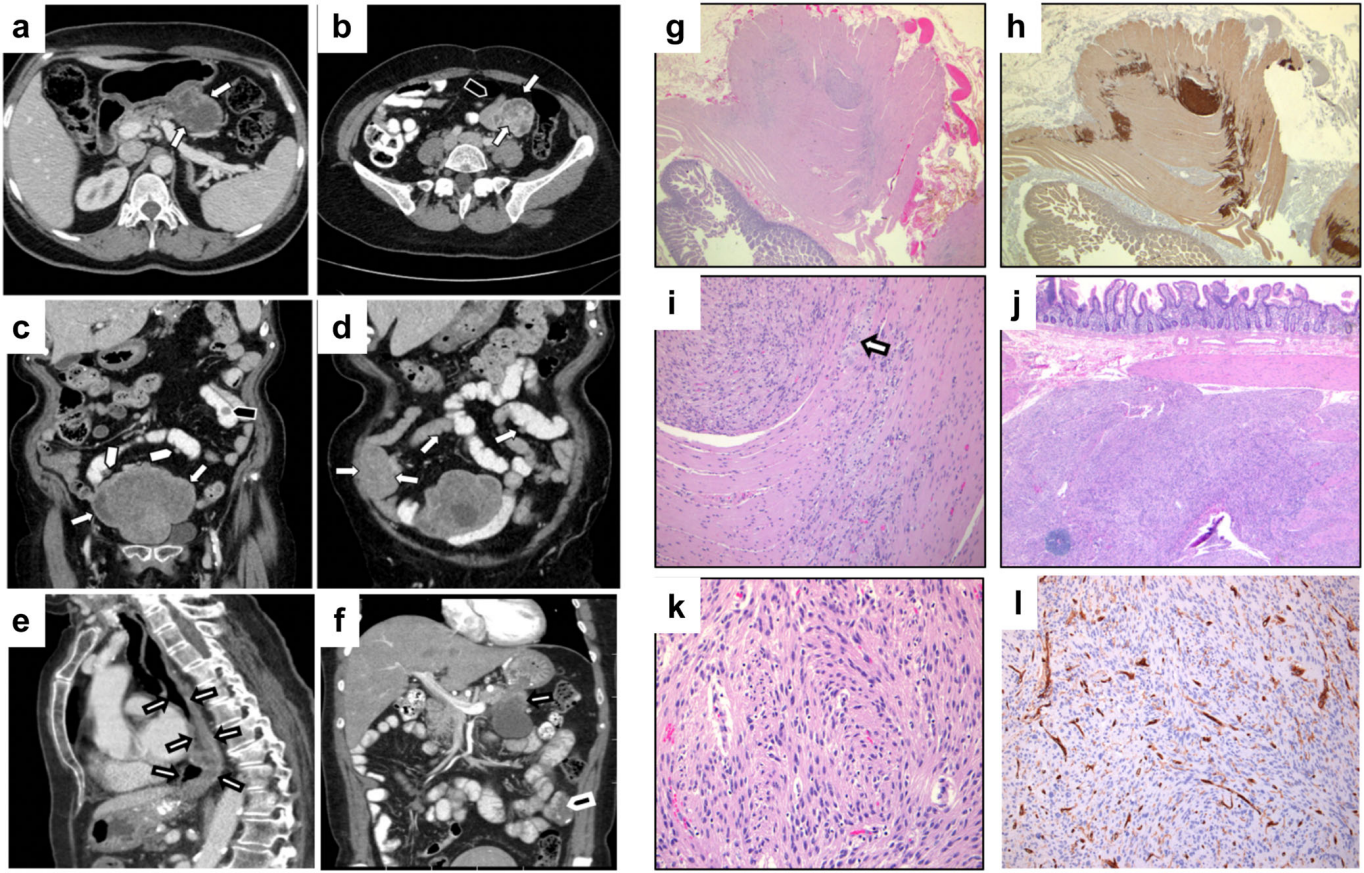
Radiologic presentation of multifocal GIST in proband (a, b) and probands mother (c–f) with histologic features (g–l). **a** Axial contrast-enhanced CT image through the upper abdomen demonstrates a lobulated exophytic solid mass arising from the lesser curvature of the stomach (arrows). The mass is entirely exoenteric, without significant intraluminal component. **b** Axial contrast-enhanced CT image through the lower abdomen demonstrates a second lobulated exophytic solid mass (arrows) arising from the distal jejunum (black tab). The mass is intimately associated with the adjacent jejunum and lies within the small bowel mesentery. **c** Coronal contrast-enhanced CT image demonstrates a dominant mesenteric heterogeneous solid mass in the lower abdomen, likely arising exophytically from the distal ileum (arrows). An intraluminal nodule is seen in the jejunum on the left side of the abdomen (black tab). Other small exophytic small bowel nodules are also present (white tabs). **d** Coronal contrast-enhanced demonstrates further numerous small bowel lesions (arrows). **e** Sagittal contrast-enhanced image demonstrates achalasia with a dilated esophagus (arrows) due to hypertrophy of the interstitial cells of Cajal. **f** Axial contrast-enhanced CT image through the upper abdomen demonstrates a lobulated exophytic solid mass arising from the lesser curvature of the stomach (arrows). The mass is entirely exoenteric, without significant intraluminal component. **g** Low power image (12.5×, H&E) showing hyperplasia of interstitial cells of Cajal along the nerve plexuses of the small bowel and a microscopic tumorlet. **h** Immunohistochemical study for CD117 highlights the GIST tumorlet and hyperplastic interstitial cells of Cajal. **i** Higher power view of tumorlet and hyperplastic interstitial cells of Cajal adjacent to the small mural ganglion (H&E, 100×), (arrow indicates ganglion). **j** Macroscopic GIST infiltrating the wall of the small bowel (H&E, 25×). **k** High power image of GIST showing classic spindle cell morphology, with fascicular architecture, fibrillary, eosinophilic cytoplasm, and blunt-ended, oval nuclei (H&E, 200×). **l** Medium power image of GIST showing largely negative CD34 in tumor cells, with strong positivity in blood vessels (100×).

The proband (III2), a 53-year-old female of Italian descent, was evaluated at the Sinai Health System, Toronto, after she presented with two months of bloating and vague abdominal pain. Abdominal computed tomography (CT) imaging revealed two exophytic masses with evidence of necrosis; a large 5.8 cm × 6 cm mass along the posterolateral aspect of the lesser curvature of the stomach (Fig. 1a) and a single lobulated mass, 4 cm × 3.8 cm in the proximal jejunum (Fig. 1b). Pathologic and immunohistochemical analysis demonstrated positivity for DOG1 and CD117 (KIT), negative CD34 staining. The gastric tumor demonstrated DOG1 and CD117 (KIT) positivity, with focal CD34 staining. The diagnosis was consistent with GIST for both tumors on final pathology. Physical examination revealed no signs of nevi, lentigines, nodules, pigmentation defects, or stigmata for neurofibromatosis type 1. Past medical history was significant for multiple uterine fibroids, ovarian cysts, and vertebral body hemangioma.

The proband’s 83-year-old mother (II4) had an incidental finding of a small bowel mesenteric mass. She had a 6-month history of weight loss due to esophageal achalasia previously requiring dilatations. Past medical history was significant for frequent urticarial episodes, multiple drug allergies, and chronic constipation. Previous surgery included a total abdominal hysterectomy and bilateral salpingo-oophorectomy for fibroids. CT of the abdomen revealed two enhancing small bowel masses, including an enhancing nodule in the proximal jejunum and an enteric enhancing mass in the distal ileum/ proximal ileum (Fig. 1c, d). There were also large lower abdominal mesenteric masses that contained coarse calcifications. She underwent laparotomy where a 15 cm × 15 cm small bowel lobulated mesenteric GIST, a protruding second 5 cm × 4 cm mass, and numerous (>50) smaller GISTs were found along the length of the small bowel. Pathology revealed a background of diffuse hyperplasia of the interstitial cells of Cajal, numerous small tumorlets, and typical spindle cell morphology with low mitotic activity (Fig. 1f–j). Immunohistochemical studies showed that the tumors expressed CD117 (KIT), DOG-1, and SMA and were largely negative for CD34, S100 protein, and desmin, consistent with a pathological diagnosis of GIST (Fig. 1l).

The proband and her mother were started on imatinib in the preoperative setting but were found to be intolerant to the drug and manifested a severe diffuse macular papular rash after 8 weeks of therapy. In the proband (patient III2), the lesions coalesced into erythematous plaques on her trunk, upper and lower extremities, interdigital folds, and perianal region, with a hyperkeratotic and scaling appearance to some lesions. Patient II4 had accompanying periorbital and lower extremity edema, along with distal upper limb paresthesia. Skin biopsy confirmed lichenoid dermatitis with lymphoeosinophilic infiltrate consistent with a drug-mediated skin reaction. This was consistent with a CTCAE Grade 3 event for both the proband and her mother. In both relatives, imatinib was withheld, and symptoms resolved with steroid treatment. The tumor responses to imatinib for the proband and her mother were consistent with stable disease. The proband showed minimal interval reduction in the size of the stomach lesion after 3 months that stabilized on follow-up imaging consistent with stable disease. Following imatinib, she underwent resection of the tumors in the stomach and small bowel. The proband has no disease recurrence 11 years from diagnosis on active surveillance. The proband’s mother (II4) also demonstrated stable disease on post-treatment imaging. Imaging was performed earlier than planned due to drug-mediated skin reactions. The patient’s mother underwent small bowel resections for her dominant and symptomatic smaller lesion.

The daughter of the proband (IV3), a 34-year-old female, has been asymptomatic and reported no gastrointestinal tract symptoms on the last follow-up on March 2, 2021. She described intermittent skin manifestations and urticaria, and her upper limbs demonstrated numerous dark brown lentigines. A skin biopsy of a bothersome facial lesion that transiently filled with fluid revealed a diagnosis of cutaneous mastocytosis. An annular lesion on her upper back was also biopsy-proven to be consistent with the annular lichen planus. There was a prior history of a dermatofibroma removed from the left arm. The proband’s sister (III-3) has the *KIT* mutation but appears not affected at 58 years of age. Annual surveillance with CT enterography has continued on these family members without any detection of GIST. There is no standard approach to surveillance for surgically treated patients and/or a high-risk family member, but generally, follow-up schedules are tailored to the risk of recurrence. CT scan is the preferred modality despite radiation exposure, and in a family with syndromic GIST, we have opted for 6-monthly imaging for an extended period of at least 10 years in those affected and baseline scans with annual reviews for family members harboring the known mutation.

### Gene panel and whole-genome sequencing identified candidate germline variants of interest in KIT and MSR1 genes

**Fig. 2:**
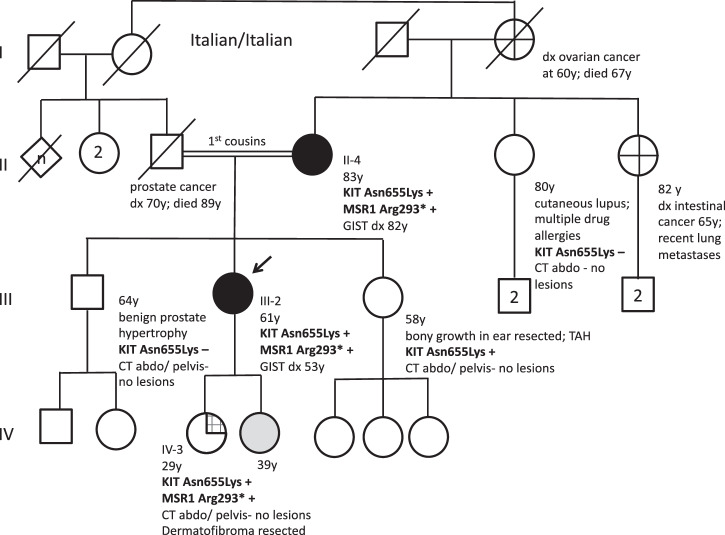
Three generational pedigree. Square, male; circle, female; strikethrough, deceased; white fill, no relevant disease; gray fill, declined assessment; black fill, GIST; grid fill, mastocytosis; cross-hatch fill, suspected GIST. The current age, age of tumor diagnosis, and age of death provided were known. Germline Asn655Lys KIT positive (+) and negative (−) genetic test results indicated. Arg293* MSR1 positive (+) genetic test results indicated. Definitions: ‘n’ refers to the presence of other relatives not specified; ‘2’ indicates two sisters or two children. Abbreviations: TAH total abdominal hysterectomy. There were no relevant familial conditions, including neurofibromatosis type 1, and cancers, including melanoma, breast carcinoma, leukemia, lymphoma, and non-GIST stromal tumors, sporadically associated with *KIT* carriers^3^.

**Fig. 3:**
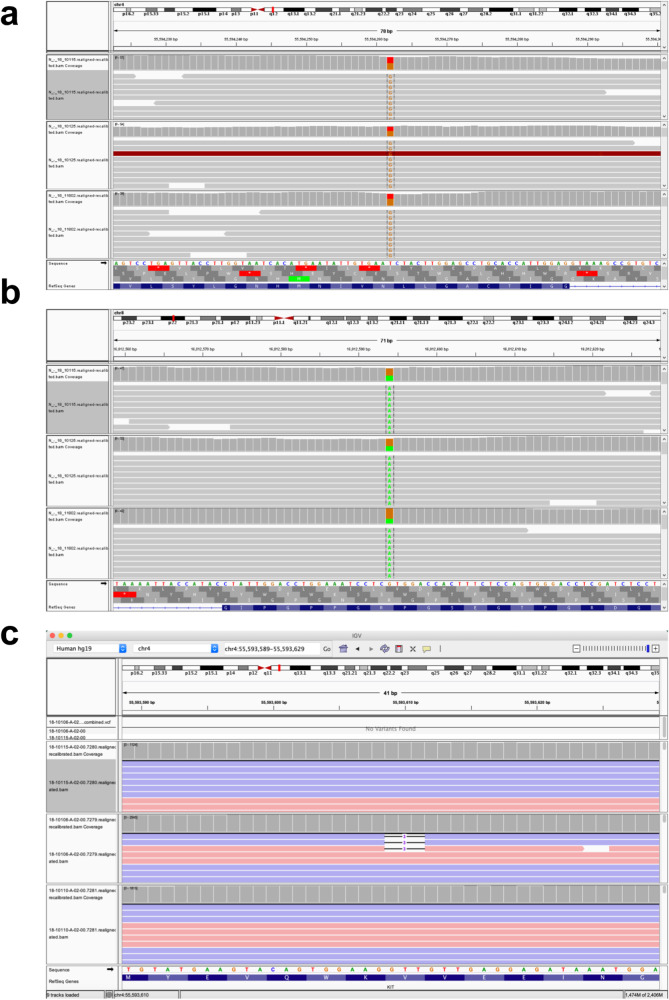
WGS of the germline. **a** *KIT* c.1965T>G variant was detected in all three generations of the kindred. **b** Shared germline stopgain variant in the MSR1 gene. IGV screenshot depicting the c.877 C > T:p.Arg293* variant in *MSR1*, predicted to result in a stop gain mutation and truncated non-functional protein product. **c** IGV screenshot depicting hotspot KIT c.1679_1681del (p.Val560del) variant detected at 1.1% VAF in one of patient II4’s tumors.

The proband, her mother, and her daughter all underwent WGS of germline DNA. Our analysis focused on the identification of pathogenic (or likely pathogenic) variants shared in the germline across multiple generations. A germline variant in *KIT* was detected (c.1965T>G; p.Asn655Lys) rs105751908 (NM_000222.2) in both the proband and her mother (Fig. 3a). Targeted testing of the same variant in *KIT* was found in the granddaughter not affected by GIST (IV3). Nonaffected relatives underwent germline gene panel analysis for the KIT variant if consent was provided (Fig. 2). All *KIT* variants found were heterozygous. Assessment of WGS data revealed also detected a stopgain variant in the *MSR1* gene in all three generations of the kindred as a candidate germline variant of interest. The variant, located in exon 6 (c.877 C > T:p.Arg293*; rs41341748; chr8: 16012594), is predicted to result in a truncated non-functional protein product (Fig. 5a). We did not identify mutations associated with other germline GIST syndromes, including *NF1*, *SDH* genes or *PDGFRA*.

### Tumor genomic analysis reveals minimal somatic mutational events

Both whole-genome and panel-sequencing reported very low mutational burden in both the proband and her mother (<1mut/Mb). From WGS, only 6–7 coding changes were found in the mother’s two tumors, and 2-3 coding changes in the proband’s two tumors, none of which were shared. We identified the germline *KIT* and *MSR1* variants in the somatic sequencing analysis for the proband and the proband’s mother. For the proband, somatic sequencing identified a variant allele fraction of 58% (*KIT*) and 63% (*MSR1*) within the one tumor and 61% (*KIT*) and 50% (*MSR1*) within the second tumor analyzed. For the proband’s mother, somatic sequencing demonstrated variant allele fractions of 47% (*KIT*) and 51% (*MSR1*) within one tumor and 55 and 57% within the second tumor analyzed for *KIT* and *MSR1,* respectively. A somatic hotspot variant, c.1679_1681del; p.Val560del, in the *KIT* gene was acquired in one of two tumors from patient II4 but was detected at a variant allele fraction of 1.1% (Fig. 3c). This somatic *KIT* variant was not detected in WGS, likely due to lower coverage that may be related to preservation, extraction and processing factors, that can all influence VAF detected.

**Fig. 4:**
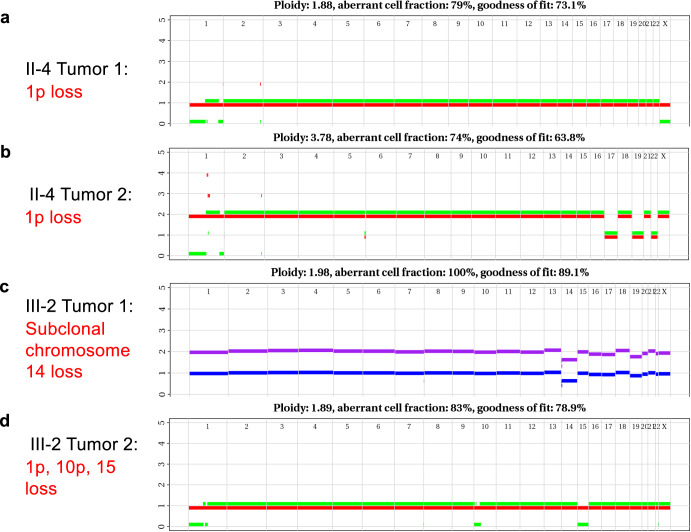
Structural variants seen in the prob and and her mother. **a** Chromosome 1p loss was seen in the proband’s mother (patient II4) in tumor 1 and tumor 2 (**b**) with an aberrant cell fraction of 79 and 74%, respectively. **c** Tumor 1 of the proband (patient III2) exhibited subclonal chromosome 14 loss, and Tumor 2 of patient III2 demonstrated chromosome 1p, 10p, and 15 loss (**d**) with aberrant cell fractions of 100 and 83%, respectively.

Previous reports have suggested that deletions in chr14q, 22q, 1p, and 15q are associated with progression from microGISTs to malignant GISTs. We used the WGS data to detect copy number changes in our patient tumors. We found deletion of chromosome 1p shared across both of the mother’s tumor samples (Fig. 4a). The proband’s two tumors had unique copy events, which included 1p, 10p, and 15 loss in one tumor, and subclonal loss of chromosome 14 in the other tumor (Fig. 4b).

## Discussion

**Fig. 5:**
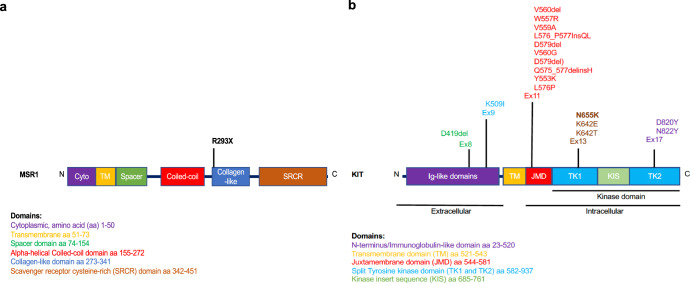
Germline variants associated with GIST. **a** MSR1 germline variant and associated predicted protein product. MSR1 protein and associated domains with R293X nonsense mutation encoded by *MSR1* c.877 C > T identified in the kindred predicted to result in truncated protein product. **b** GIST-associated KIT germline mutations reported in the literature^3^, including the current study and the associated affected residue and protein domains.

We report a kindred with multiple generations affected with multifocal GIST and intolerance to preoperative imatinib. Through the WGS of the tumor, a germline variant in *KIT*, c.1965T>G; p.Asn655Lys (p.N655K) was found. This variant is not present in population databases (ExAC, gnomAD) but has been reported in jejunal GIST, acral melanoma, and acute myeloid leukemia as a somatic variant^11–14^. p.N655K resides in the kinase domain and has been shown to induce ligand-independent activation of the receptor and downstream signaling pathways, which has been shown to be inhibited by imatinib in patients with melanoma and/or GIST^11,12^. Imatinib has been shown to be highly effective in sporadic GISTs, with 93% of patients from the literature with *KIT* exon 13 mutations (almost all with a p.K642E mutation) achieving at least stable disease^1^. Family members affected with GIST and harboring this germline variant demonstrated a clinical phenotype that appears consistent with the role of *KIT* in mast cells, melanocytes and interstitial cells of Cajal. The affected members of the kindred who have developed multifocal GIST demonstrate gut motility dysfunction (II-4) and multiple drug intolerances that include imatinib. Other isolated relatives carrying the *KIT* germline variant have manifested cutaneous lesions- such as cutaneous mastocytosis and dermatofibroma (IV-3), and two other relatives (I-4) and (II-6) had histories suggestive of GISTs (Fig. 2). A graphical summary of germline KIT variants and the affected residues and protein domains is depicted in Fig. 5b.

*KIT* variants cause a loss of the inhibitory regulatory effect of the juxtamembrane domain of *KIT* on the tyrosine kinase pathway and other downstream pathways^15^. The mechanism of imatinib is to inhibit this downstream signaling cascade. Clinical reports have shown the relationship between exon-specific *KIT* variants and response to imatinib, including better sensitivity to imatinib with exon 11 deletions, than with other exon 11 variants such as insertions or substitution variants, exon 9 variants and wild-type genotypes^16–18^. In this respect, with further characterization of specific variants, genomic sequencing of the GIST tumor can be employed within the armamentarium of the treating clinician to help determine the most efficacious treatment strategy, as it is certainly possible that other gene variants may play an important role in modulating treatment efficacy and may come to light with future studies as additional molecular biomarkers in GIST. There are limited reports describing the variable efficacy of imatinib with partial response and stability of GIST lesions in kindreds harboring a germline exon 13 *KIT* variant^1,19,20^. Importantly, failure of imatinib therapy can be seen with the development of secondary resistance mutations in up to 90% of patients with GIST, at specific hotspot locations in the ATP binding pocket encoded by exons 13 and 14 and in the activation loop encoded by exons 17 and 18^18^. Interestingly, the hotspot within exon 13, encoding the V654 mutation, has been shown to demonstrate resistance to imatinib^18,21^. The role of other mutations within the ATP binding pocket is not well-characterized in the primary setting, given the rarity of primary mutations in this region. However, studies of newly diagnosed jejunal GIST with p.N655K mutation have shown ligand-independent autophosphorylation and sensitivity to imatinib treatment^11^. Studies in melanoma have demonstrated variable response rates of imatinib treatment based on mutation, with data demonstrating only a 33% response rate within patients with exon 13 mutations^22^. Patients with KIT N599K mutations were not specifically assessed in this dataset however^22^. In this report, the authors acknowledge that earlier imaging prompted by drug-mediated reactions and the short course of therapy may explain the lack of meaningful response to imatinib, although it is not clear why the proband and her mother demonstrated intolerance to the drug in the form of drug-limiting skin toxicity. Although not available at the time of treatment in 2011 for the cases within this report, the inhibitor nilotinib may represent a good candidate for next-line treatment in such cases given its limited cross-reactivity with imatinib^23^, although further studies are required to elucidate its potential predicted tumor response in this and similar clinical scenarios. Taken together, further validation is needed in germline tumor models to elucidate the significance of p.N655K when making mutation-informed treatments in patients with GIST.

WGS germline variant profiling also revealed an *MSR1* stop gain variant in exon 6 (c877C>T; p.Arg293*) rs41341748 shared between patients over 3 generations of this family. *MSR1* at 8p22 encodes the class A macrophage scavenger receptor which is specific for glycoproteins primarily expressed on tissue macrophages and dendritic cells and linked to inflammatory and pathological processes^24,25^. Germline variants have been found in the *MSR1* gene and have been associated with susceptibility to hereditary prostate cancer^26–28^ and in patients with upper gastrointestinal pathologies such as Barrett’s esophagus and esophageal adenocarcinoma^24^. Germline variants of *MSR1* have also been identified rarely in cases of hereditary diffuse gastric cancer^29^ and have shown function in M2 macrophage polarization, with emerging functions being seen in tumor-associated macrophages^30,31^. Three isoforms exist via alternative splicing of this gene, with isoforms 1 and 2 acting as functional receptors and able to modulate endocytosis of modified low-density lipoproteins (m-LDL)^32^, and isoform 3, a truncated isoform, unable to internalize m-LDL and interestingly capable of acting in a dominant negative fashion when co-expressed with isoforms 1 and 2^31,32^. Previous reports have shown that CD204 (MSR1) has been found to be upregulated by imatinib treatment^33^. In leukemia stem cells, MSR1 has been identified to harbor tumor suppressor gene function^34^. Although the role of *MSR1* mutation remains largely unknown in this family, it demonstrates an interesting candidate gene for further investigation.

The somatic genome for patients III2 and II4 did not show any shared coding change variants between tumors. However, patient II4’s tumors shared a chr1p loss previously implicated in malignant GIST progression. While patient III2’s tumors had no shared copy number events, each had copy specific number events (1p, 10p, 14 loss) also implicated in malignant GIST. These results suggest that these multifocal tumors may arise from shared ancestral clones, which then evolve in parallel or as independent primary tumors. Deeper sequencing of these tumors and of tumors from multiple foci within these patients will further elucidate how these tumors evolve.

Our study is the first report that we are aware of demonstrating the genomic characterization of a family with multigenerational GIST. Herein, we describe a rare germline variant in *KIT* c.1965T>G; p.Asn655Lys which was harbored by affected family members. A variant in a candidate gene, *MSR1*, was also found to be co-segregating with the *KIT* variant and may be contributory to the family’s phenotype, although this requires further validation. Further characterization of each of these germline variants and their role in this phenotype may shed light on the molecular mechanisms leading to the development of GIST.

## Methods

### Ethics statement

A genetic etiology was considered in this family due to the younger age of onset and the presence of multifocal GIST over two generations (see pedigree, Fig. 2). The patients provided written informed consent with research ethics board approval at the University Health Network / Princess Margaret Cancer Centre, Mount Sinai Hospital and The Hospital for Sick Children (SickKids) for the retrieval of relevant medical data for research purposes, consent for publication and case reporting, consent for data deposition in a public database and to determine the basis of underlying hereditary cancer susceptibility, complying with all relevant ethical regulations including the Declaration of Helsinki. The proband (III2) and her mother’s (II4) tumors were sequenced through the SickKids Cancer Sequencing Program with Research Ethics Board approval at The Hospital for Sick Children (Toronto, Ontario, Canada), KiCS, available at https://www.kicsprogram.com.

### KiCS tumor-normal panel sequencing

KiCS sequencing was performed on banked frozen tumor tissue (×2) located at gastric and small bowel sites from the proband, III2, and tissue from two tumors resected from the small bowel and distal ileum from patient II4 along with a matched normal germline blood sample for each patient. The SickKids Cancer Sequencing (KiCS) gene panel is a clinically validated test that utilizes the Agilent SureSelect capture kit technology, followed by paired-end sequencing of the coding and splice site regions using the Illumina sequencing platform to sequence 15,000 exons across 880 genes including the common genes associated with familial GIST (*KIT*, *NF1*, *PDGFRA*, *SDHA*, *SDHB*, *SDHC*, *SDHD*). Enriched libraries were prepared from both tumor DNA and matched normal (blood or skin) and sequenced on Illumina HiSeq2500 sequencers running in rapid mode producing paired-end 100 base reads. Reads were aligned with Burrows–Wheeler Aligner (BWA)-MEM according to Genomic Analysis Tool Kit (GATK) best practices with coverage metrics meeting greater than 700× mean coverage, with ≥98.5% of bases above 50×, ≥95% of bases above 200× coverage, and ≥75% of bases above 500× coverage. Substitution variants were called using MuTect, with variants called above 50× coverage in tumor and normal. Somatic variants were called against the matched germline sample from the sample patient by tumor-normal subtractive analysis. Tumor mutation burden (TMB) was measured as the number of somatic variants within exonic coding regions per Megabase (Mb) of DNA (VAF > 5%, within ±10 base pairs of intron–exon junctions).

### KiCS tumor-normal whole-genome sequencing and analysis

WGS was performed on banked frozen tumor tissue (×2) located at gastric and small bowel sites from the proband, III2, and tissue from two tumors resected from the small bowel and distal ileum from patient II4 along with a matched normal germline blood sample for each patient. Sequencing was performed on Illumina HiSeqX to a minimum depth of 30×. FASTQs were aligned to hg19 using BWA-MEM (v0.78). PCR duplicates were marked with Picard (v1.1.08), with indel realignment and recalibration of base quality scores using GATK (v2.8.1). Variant calls were generated using the Genomic Analysis Tool Kit (GATK) after read alignment with the BWA. All called somatic base substitutions were filtered for quality control as previously described^35^. Briefly, we required a minimum depth of 10× in the tumor and normal with 0 reads supporting the variant in the matched normal. We also removed those variants found in a panel of normal non-neoplastic tissue sequenced (*n* = 133) and analyzed using the same methods, as well as those that failed at least 2 of 4 cutoffs for non-unique mapping (<70% of reads at locus map uniquely), multi-mapping clusters (seen in tumor and matched normal), excessively high mapping depth (vs the average of the normal chromosome) and presence in low complexity regions (DUST score > 60). WGS Mutation burden was calculated separately for SNVs, Indels, and SVs. For WGS, to calculate mutation burdens per megabase, the count of all coding and non-coding variants that passed the above QC filters was divided by a genome size of 2800 Mb. Copy number changes were detected from WGS using Battenberg V3.3.2, which takes into account both log-fold changes in sequencing depth and changes in allele fraction to provide allele-specific integer copy numbers from matched tumor-normal sequencing.

### Reporting summary

Further information on research design is available in the Nature Research Reporting Summary linked to this article.

### Supplementary information

REPORTING SUMMARY

## Data Availability

Identified germline variants were deposited in ClinVar at accession number SCV000630449. Inquiries for access to raw sequencing data can be directed to the corresponding authors for further information.

## Code availability

Code utilized for sequencing data alignment and processing is detailed within the methods section of this report utilizing publicly available published techniques and version codes described in the report.
