# Supplementary material for: Genomic and clinical characterization of a familial GIST kindred intolerant to imatinib
Source: NPJ Genom Med. 2024 Mar 27;9:24. doi: 10.1038/s41525-024-00405-z (PMC10973472; doi:10.1038/s41525-024-00405-z)
Supplement: Supplementary file 1 — REPORTING SUMMARY [file 41525_2024_405_MOESM1_ESM.pdf]

nature portfolio

Corresponding author(s): Rebecca Gladdy, Raymond Kim

Last updated by author(s): 2024/02/29

Reporting Summary

Nature Portfolio wishes to improve the reproducibility of the work that we publish. This form provides structure for consistency and transparency in reporting. For further information on Nature Portfolio policies, see our Editorial Policies and the Editorial Policy Checklist.

Statistics

For all statistical analyses, confirm that the following items are present in the figure legend, table legend, main text, or Methods section.

n/a	Confirmed	
☒	☐	The exact sample size (*n*) for each experimental group/condition, given as a discrete number and unit of measurement
☒	☐	A statement on whether measurements were taken from distinct samples or whether the same sample was measured repeatedly
☒	☐	The statistical test(s) used AND whether they are one- or two-sided<br>*Only common tests should be described solely by name; describe more complex techniques in the Methods section.*
☒	☐	A description of all covariates tested
☒	☐	A description of any assumptions or corrections, such as tests of normality and adjustment for multiple comparisons
☒	☐	A full description of the statistical parameters including central tendency (e.g. means) or other basic estimates (e.g. regression coefficient) AND variation (e.g. standard deviation) or associated estimates of uncertainty (e.g. confidence intervals)
☒	☐	For null hypothesis testing, the test statistic (e.g. *F*, *t*, *r*) with confidence intervals, effect sizes, degrees of freedom and *P* value noted<br>*Give P values as exact values whenever suitable.*
☒	☐	For Bayesian analysis, information on the choice of priors and Markov chain Monte Carlo settings
☒	☐	For hierarchical and complex designs, identification of the appropriate level for tests and full reporting of outcomes
☒	☐	Estimates of effect sizes (e.g. Cohen's *d*, Pearson's *r*), indicating how they were calculated

Our web collection on statistics for biologists contains articles on many of the points above.

Software and code

Policy information about availability of computer code

Data collection	Sequencing reads were aligned using Burrows-Wheeler Aligner (BWA)-MEM v0.78 according to the Genomic analysis tool kit (GATK v2.8.1)vbest practices, substitution variants were called using MuTect. Copy number changes were detected from whole-genome sequencing data using Battenberg v3.3.2
Data analysis	Sequencing reads were aligned using Burrows-Wheeler Aligner (BWA)-MEM v0.78 according to the Genomic analysis tool kit (GATK v2.8.1)vbest practices, substitution variants were called using MuTect. Copy number changes were detected from whole-genome sequencing data using Battenberg v3.3.2

For manuscripts utilizing custom algorithms or software that are central to the research but not yet described in published literature, software must be made available to editors and reviewers. We strongly encourage code deposition in a community repository (e.g. GitHub). See the Nature Portfolio guidelines for submitting code & software for further information.

Data

Policy information about availability of data

All manuscripts must include a data availability statement. This statement should provide the following information, where applicable:

- Accession codes, unique identifiers, or web links for publicly available datasets
- A description of any restrictions on data availability
- For clinical datasets or third party data, please ensure that the statement adheres to our policy

Identified germline variants were deposited in ClinVar at accession number SCV000630449. Inquiries for access to raw sequencing data can be directed to the corresponding authors for further information.

Research involving human participants, their data, or biological material

Policy information about studies with human participants or human data. See also policy information about sex, gender (identity/presentation), and sexual orientation and race, ethnicity and racism.

Reporting on sex and gender	The case report herein includes the sex of the proband, her mother and family members for the purposes of the familial kindred and its description.
Reporting on race, ethnicity, or other socially relevant groupings	The case report herein includes the Italian heritage of the proband, her mother and the familial kindred for descriptive reporting purposes.
Population characteristics	See below
Recruitment	A genetic etiology was considered in this family due to the younger age of onset and the presence of multifocal GIST over two generations
Ethics oversight	Ethics statement<br>A genetic etiology was considered in this family due to the younger age of onset and the presence of multifocal GIST over two generations (see pedigree, Figure 2). The patients provided written informed consent with research ethics board approval at University Health Network / Princess Margaret Cancer Centre, Mount Sinai Hospital and The Hospital for Sick Children (SickKids) for the retrieval of relevant medical data for research purposes, consent for publication and case reporting, consent for data deposition in a public database and to determine the basis of underlying hereditary cancer susceptibility. The proband (III2) and her mothers' (II4) tumors were sequenced through the SickKids Cancer Sequencing Program with Research Ethics Board approval at The Hospital for Sick Children (Toronto, Ontario, Canada), KiCS, available at https://www.kicsprogram.com.

Note that full information on the approval of the study protocol must also be provided in the manuscript.

Field-specific reporting

Please select the one below that is the best fit for your research. If you are not sure, read the appropriate sections before making your selection.

☒ Life sciences ☐ Behavioural & social sciences ☐ Ecological, evolutionary & environmental sciences

For a reference copy of the document with all sections, see nature.com/documents/nr-reporting-summary-flat.pdf

Life sciences study design

All studies must disclose on these points even when the disclosure is negative.

Sample size	The patient, her mother were the affected family members who developed GIST and as such, they were included in somatic tumor analyses.
Data exclusions	No data was excluded.
Replication	Sequencing was performed with whole-genome methods and cancer sequencing panels.
Randomization	Not applicable.
Blinding	Not applicable.

Reporting for specific materials, systems and methods

We require information from authors about some types of materials, experimental systems and methods used in many studies. Here, indicate whether each material, system or method listed is relevant to your study. If you are not sure if a list item applies to your research, read the appropriate section before selecting a response.

Materials & experimental systems

n/a	Involved in the study
☒	☐ Antibodies
☒	☐ Eukaryotic cell lines
☒	☐ Palaeontology and archaeology
☒	☐ Animals and other organisms
☒	☐ Clinical data
☒	☐ Dual use research of concern
☒	☐ Plants

Methods

n/a	Involved in the study
☒	☐ ChIP-seq
☒	☐ Flow cytometry
☒	☐ MRI-based neuroimaging

Plants

Seed stocks	Not applicable.
Novel plant genotypes	Not applicable.
Authentication	Not applicable.
